# Value of whole-process quality control management in central sterile supply department nursing practice and nosocomial infection prevention

**DOI:** 10.12669/pjms.42.3.13511

**Published:** 2026-03

**Authors:** Fengjuan Wang, Na Zhao

**Affiliations:** 1 Fengjuan Wang Sterilization Supply Center, Affiliated Hospital of Hebei University, Baoding 071000, Heibei, China; 2 Na Zhao Endoscopic Diagnostic and Treatment Center, Affiliated Hospital of Hebei University, Baoding 071000, Heibei, China

**Keywords:** Central sterile supply department, Disinfection and sterilization, Nursing management, Nosocomial infection prevention, Qualification, Whole-process quality control management

## Abstract

**Objective:**

To evaluate the application value of whole-process quality control(WPQC) management in central sterile supply department(CSSD) nursing management and nosocomial infection prevention.

**Methodology:**

This was a retrospective study. A total of 300 medical instrument packs (including luminal devices) processed by the Sterilization Supply Center of Affiliated Hospital of Hebei University between January and June, 2025 were selected and randomly assigned, using a random number table, into a control group (*n=* 150) and an observation group (*n =* 150). The control group received standard CSSD nursing management, while the observation group was managed using the WPQC model. Sterilization qualification rates, error rates, time consumption at each processing stage, nurse compliance with operational standards, and the incidence of related nosocomial infections were compared between the two groups.

**Results:**

Compared with the control group, the observation group demonstrated significantly higher qualification rates for recovery, cleaning, disinfection, sterilization, packaging, and distribution(all *P<* 0.05). The overall error rate was significantly lower in the observation group than in the control group(*P<* 0.05). The observation group also had shorter processing times for cleaning, packaging, and sterilization, with nurses showing better compliance with operational standards (*P<* 0.05, respectively). Furthermore, the overall incidence of related nosocomial infections was significantly reduced in the observation group(*P<* 0.05).

**Conclusion:**

The implementation of WPQC management in CSSD nursing management may substantially improve qualification rates across all stages of medical instrument disinfection and sterilization, reduce error and infection rates, shorten processing time, and enhance nurses’ adherence to operational standards.

## INTRODUCTION

The central sterile supply department (CSSD) is a pivotal unit in hospitals, responsible for the recovery, cleaning, disinfection, sterilization, storage, and distribution of all reusable medical instruments. Its performance is directly linked to medical safety and patient recovery outcomes, serving as both a critical checkpoint and the first line of defense in nosocomial infection prevention and control.^1^-^3^ Traditional nursing management models primarily emphasize final quality inspection, leaving gaps in supervision between stages. As a result, they fail to provide meticulous and standardized oversight across the entire processing cycle of medical instruments.

These deficiencies pose potential safety risks that may lead to nosocomial infections, such as surgical site infections or catheter-related bloodstream infections, thereby increasing patient suffering and exacerbating the healthcare burden.^4^-^6^ With the rapid advancement of medical technology in recent years, the use of precise, complex instruments has become increasingly common, imposing stricter demands on CSSD operations.^7^ Against this backdrop, WPQC management has been introduced as a modern, systematic approach to CSSD management. This model emphasizes the establishment of clear quality standards, standardized procedures, and designated responsibilities at every stage, from instrument recovery to distribution.

In addition, the integration of information-based traceability systems enables real-time monitoring and data-driven management of the entire process, ensuring that quality issues can be promptly identified, traced, intervened upon, and corrected.^8^,^9^ Building upon these considerations, a scientific evaluation of WPQC management was conducted by comparing it with conventional management practices in terms of qualification rates, operational compliance, processing efficiency, and the incidence of nosocomial infections, aiming to provide robust empirical evidence on the comprehensive value and practical effectiveness of WPQC management in enhancing CSSD nursing management, ensuring instrument safety, and preventing nosocomial infections, thereby offering meaningful guidance for hospital quality management.

## METHODOLOGY

This was a retrospective study. A total of 300 medical instrument packs (including luminal instruments) processed by the Sterilization Supply Center of Affiliated Hospital of Hebei University between January and June 2025 were selected. Using a random number table, the packs were divided into two groups: the control group (*n=* 150) and the observation group (*n=* 150). The study involved 20 staff members, including three males and 17 females, aged 25-48 years, with a mean age of 33.10 ± 3.99 years. Among them, four had a junior college education, and 16 held a bachelor’s degree. Statistical analysis showed no significant differences between the two groups in instrument categories and departmental sources (both *P >* 0.05) Table-I.

**Table-I:** Comparison of general characteristics between the two groups.

Group	Category			Source			
	Scissors, n(%)	Hemostatic forceps, n(%)	Luminal instruments, n(%)	Internal medicine, n(%)	Surgery, n(%)	ICU, n(%)	Others
Observation (*n* = 150)	52(34.67)	42(28)	56(37.33)	50(33.33)	38(25.33)	23(15.33)	39(26)
Control (*n* = 150)	54(36)	43(28.67)	53(35.33)	51(34)	39(26)	25(16.67)	35(23.33)
*t/χ*² value	0.132			0.322			
*P*-value	0.936			0.956			

### Ethical approval:

The study was approved by the Institutional Ethics Committee of Affiliated Hospital of Hebei University (No.: HDFYLL-KY-2024-105; date: May 06, 2024), and written informed consent was obtained from all participants.

### Inclusion criteria:

- All instruments originated from our hospital and conformed to national and industry standards for medical devices.
- All instruments managed by the same team of staff with more than one year of work experience.
- Informed consent from all staff involved.

### Exclusion criteria:

- Instruments that were damaged or with missing identification information.
- Externally sourced instruments.
- Disposable instruments.

The control group was managed using the standard CSSD nursing management model, which mainly included the following procedures: recovery and preliminary classification of instruments according to established workflows; standard cleaning and disinfection operations; inspection of instruments after cleaning and disinfection; packaging and storage; and distribution of instruments following the “first-in, first-out” principle, in order to maintain standardized management and continuous improvement.

The observation group was managed using a whole-process quality control (WPQC) management model, which centers on risk management and establishes a refined management system covering the entire chain of medical instrument processing-from recovery, classification, cleaning, disinfection, inspection, packaging, sterilization, storage, and distribution to final traceability. This model incorporates standardized operating procedures (SOPs), critical control points (CCPs), and an information-based traceability system to ensure clear accountability, measurable processes, and traceable quality at every stage. The specific implementation pathway was as follows:

### Organizational structure and responsibility allocation:

A WPQC management team was established, consisting of the head nurse, quality control officers, and sterilization engineers. The team was responsible for developing tailored SOPs for different categories of instruments based on the Guidelines for Management of Hospital Central Sterile Supply Department and manufacturers’ instructions. Examples included *Key Quality Control Points for Endoscopic Instrument Cleaning* and *Procedures for Handling External Instruments*. The team also conducted regular staff training and competency assessments to ensure that every staff member was fully aware of their responsibilities and quality requirements.

### Environmental zoning and precise parameter control:

The CSSD work areas were strictly divided into disinfection, inspection/packaging/sterilization (including separate rooms for dressing preparation or packaging), and sterile storage zones. These areas were physically separated with clearly defined access routes. Automatic temperature and humidity monitoring systems were employed to maintain the working environment at 20-22°C and 50% ± 5% relative humidity, with daily records verified and signed. Weekly microbiological sampling was performed on work surfaces, equipment, and air. If results exceeded acceptable limits, an immediate re-cleaning and disinfection protocol for the environment was activated.

### Process quality monitoring and staff competency development:

An adenosine triphosphate bioluminescence detector was introduced to conduct quantitative spot checks of instruments after cleaning, with the qualification threshold set at RLU < 50. Instruments failing to meet the standard were returned for reprocessing. Quarterly biological and chemical monitoring was performed on cleaning water and sterilizers. A personnel training archive was established, and monthly skill-based training and assessments were conducted on instrument identification, detergent preparation, and equipment operation. Only staff who passed the evaluations were permitted to continue working.

### Standardization and informatization of key operational stages:

### Cleaning stage:

Based on the degree of contamination (*e.g*., heavy blood or protein residues) and material type, explicit requirements were set for multi-enzyme cleaning solution concentration (*e.g*., 1:200), soaking time (≥ 5 minutes), and rinsing water temperature (30-45°C). Complex instruments (*e.g*., surgical drills, endoscopic sheaths) were cleaned following a standardized pathway: pre-cleaning → ultrasonic cleaning → rinsing → terminal rinsing.

### Inspection and packaging stage:

All instruments were examined under an illuminated magnifying lens, with 100% inspection of functional tips and joints to ensure the absence of blood stains, rust, or damage. Double-layer cotton or nonwoven fabric was used for packaging. Before sealing, instrument categories and quantities were verified and recorded in the system by barcode scanning. Package labels included item name, sterilization date, expiration date, and operator ID.

### Sterilization and distribution stage:

Each sterilization cycle was monitored with chemical and biological indicators, and results were automatically uploaded to the traceability system. Sterile storage racks were positioned 20-25 cm above the floor and 5-10 cm from the wall. During distribution, the system automatically identified the earliest-expiring batch to ensure compliance with the “first-in, first-out” principle. Barcode scanning at dispatch synchronized records of the receiving department, recipient, and time, thereby achieving closed-loop traceability.

### Observation indicators:

Qualification rates of disinfection and sterilization:

### Cleaning qualification rate:

Proportion of instruments with no visible contaminants (*e.g*., dirt, blood stains, rust) and an ATP bioluminescence value below the threshold (RLU < 50) after cleaning;

### Disinfection qualification rate:

Proportion of instruments achieving the required disinfection level (*e.g*., high-level disinfection) after treatment, as verified by chemical or biological indicators;

### Sterilization qualification rate:

Proportion of sterilization packs meeting all physical, chemical, and biological monitoring standards, with negative results for biological indicators; (

### Packaging qualification rate:

Proportion of instruments packaged in full compliance with standards for materials, methods, and labeling (including item name, sterilization date, expiration date, and operator ID);

### Distribution qualification rate:

Proportion of sterile items distributed with intact packaging, clear labeling, validity within the expiration period, and conformity with departmental requirements.

### Error rate:

Non-standard packaging, unclear labeling, presence of wet packs, delayed distribution, or missing instruments inside sterilization packs.

### Processing time and nurse compliance with SOPs:

### Cleaning time:

Duration from instrument recovery and classification to completion of cleaning;

### Packaging time:

Duration from post-cleaning inspection to completion of packaging and sealing;

### Sterilization time:

Total duration including loading, sterilization cycle, cooling, and unloading;

### Nurse compliance rate:

Proportion of cases in which nurses adhered strictly to SOPs across all stages of instrument processing. Incidence of related nosocomial infections: Within one month after surgery, patients in whom the study instruments were used were followed up to record nosocomial infections, including surgical site infections, catheter-related bloodstream infections, urinary tract infections, and pulmonary infections.

### Statistical analysis:

All data were analyzed using SPSS 27.0. Categorical variables were expressed as frequency (*n*) and percentage (%) and compared using the chi-square (χ²) test or Fisher’s exact test. Continuous variables were tested for normality using the Shapiro-Wilk test. The confidence interval was 95%. Data conforming to normal distribution were expressed as mean ± standard deviation (*χ̅±s*) and compared using independent-samples t tests. A *P*-value <0.05 was considered statistically significant.

## RESULTS

The observation group achieved significantly higher qualification rates in recovery, cleaning, disinfection, sterilization, packaging, and distribution compared with the control group (all *P <* 0.05) Table-II. The overall error rate was significantly lower in the observation group than in the control group (*P <* 0.05) Table-III.

**Table-II:** Comparison of disinfection and sterilization qualification rates between the two groups (n[%]).

Group	Recovery	Cleaning	Disinfection	Sterilization	Packaging	Distribution
Observation (n = 150)	150(100)	150(100)	150(100)	149(99.33)	150(100)	150(100)
Control (n = 150)	145(96.67)	146(97.33)	146(97.33)	143(95.33)	146(97.33)	145(96.67)
*χ*² value	5.085	4.054	4.054	4.623	4.054	5.085
*P*-value	0.024	0.044	0.044	0.032	0.044	0.024

**Table-III:** Comparison of error rates in instrument management between the two groups (n[%]).

Group	Non-standard packaging	Unclear labeling	Wet packs	Delayed distribution	Missing instruments	Total error rate
Observation (n = 150)	0(0.00)	0(0.00)	1(0.67)	1(0.67)	1(0.67)	3(2.00)
Control (n = 150)	5(3.33)	1(0.67)	3(2)	1(0.67)	3(2.000)	13(8.67)
*χ*² value	-	-	-	-	-	6.602
Fisher/P-value	0.060	1.000	0.622	1.000	0.622	0.010

**Fig.1:**
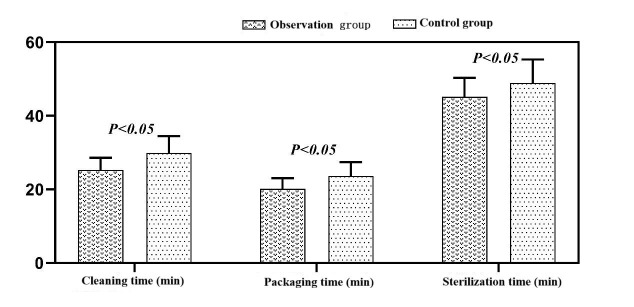
Bar chart comparison of processing times between the two groups.

The observation group also had shorter processing times for cleaning, packaging, and sterilization, with nurses showing better compliance with operational standards (*P <* 0.05, respectively) Table-IV and Fig.1. Furthermore, the overall incidence of related nosocomial infections was significantly reduced in the observation group (*P <* 0.05) Table-V.

**Table-IV:** Comparison of processing time and nurse compliance with SOPs between the two groups.

Group	Cleaning time (min)	Packaging time (min)	Sterilization time (min)	Nurse compliance, n(%)
Observation (n = 150)	25.29±3.29	20.15±2.87	45.15±5.17	149(99.33)
Control (n = 150)	29.87±4.61	23.64±3.78	48.94±6.39	136(90.67)
*t/χ*² value	9.906	9.006	5.643	11.860
P-value	<0.001	<0.001	<0.001	0.001

**Table-V:** Comparison of nosocomial infection rates between the two groups, n(%).

Group	Surgical site infection	Urinary tract infection	Respiratory infection	Other infections	Total incidence
Observation (n = 150)	1(0.67)	0(0.00)	0(0.00)	0(0.00)	1(0.67)
Control (n = 150)	4(2.67)	2(1.33)	1(0.67)	1(0.67)	8(5.33)
Fisher	0.371	0.498	1.000	1.000	0.036

## DISCUSSION

The findings of the present study demonstrated that, compared with the control group, the observation group achieved significantly higher qualification rates for recovery, cleaning, disinfection, sterilization, packaging, and distribution, while maintaining a lower overall error rate. These results indicate that applying WPQC management in CSSD nursing practice can substantially enhance qualification rates at each stage of instrument disinfection and sterilization and effectively reduce the error rate. Several factors may explain these improvements. First, the WPQC model establishes standardized operating procedures and clearly defined quality control checkpoints throughout the entire workflow, ensuring closed-loop management from recovery to distribution. The CSSD serves as the “core hub” of nosocomial infection prevention and control, and its quality of work directly affects surgical safety, patient outcomes, and the overall standard of hospital medical safety.^10^-^12^ Traditional CSSD management models are limited by fragmented processes, lagging quality control, and heavy reliance on manual experience.^13^,^14^ Fang L et al.^15^ reported that WPQC management can address these shortcomings by restructuring the management framework-establishing a dedicated quality control team, defining job responsibilities, formulating standardized operating procedures, and incorporating information-based traceability systems. This approach enables real-time monitoring, early warning, and accountability across every stage from instrument recovery to distribution, thereby shifting CSSD operations from experience-based practices toward process-oriented, data-driven, and refined scientific management.

This system-level approach minimizes the risks associated with fragmented processes and ambiguous responsibilities, thereby improving the quality of end products. Second, the incorporation of an information-based traceability system allows real-time monitoring and immediate interception of abnormalities, shifting the focus of quality control from “end-point inspection” to “process management,” and effectively preventing the circulation of noncompliant products. Third, the establishment of a dedicated quality control team, coupled with regular staff training and competency assessments, not only improves technical proficiency but also reinforces a sense of accountability, further ensuring standardization and rigor in practice. Together, these measures contributed to improved cleaning and packaging qualification rates and a reduction in error occurrence. Similarly, Van der Starre CM et al.^16^ reported that WPQC management in CSSD significantly optimizes the quality of disinfection and sterilization of medical instruments. Their findings showed improved qualification rates, reduced operational errors, and enhanced nursing quality, ultimately contributing to greater clinical satisfaction.

Furthermore, this study found that the observation group demonstrated significantly shorter cleaning, packaging, and sterilization times, as well as a higher rate of adherence to standardized operating procedures compared with the control group. These findings suggest that WPQC management in CSSD not only improves disinfection and sterilization quality but also enhances workflow efficiency and nurse compliance with operating standards. The favorable observations may be explained by the following factors: First, the establishment of explicit and detailed SOPs decomposed complex tasks into clear, standardized steps, reducing redundancy and preventing time wasted due to procedural ambiguity, thereby markedly shortening processing time at each stage. Second, targeted training and regular competency assessments enabled nurses in the observation group to perform instrument handling more quickly and accurately, while stringent supervisory mechanisms ensured adherence to operating standards, achieving both efficiency and quality simultaneously. These conclusions are consistent with the findings of Zhang R et al.^17^ further highlighted that nosocomial infections not only increase patient suffering but also significantly prolong hospitalization and exacerbate the economic burden of care. Importantly, the incidence of nosocomial infections serves as the ultimate endpoint indicator for evaluating CSSD performance. The quality of instrument cleaning, disinfection, and sterilization directly determines patient safety; any lapse in these processes can render instruments a vehicle for pathogen transmission, resulting in severe consequences such as surgical site infections or catheter-related bloodstream infections. In this study, the observation group had a significantly lower incidence of nosocomial infections compared with the control group, indicating that WPQC management can effectively reduce infection risk in clinical practice.

The underlying mechanisms may include the establishment of a robust “aseptic defense line” by transforming fragmented disinfection and sterilization steps into a closed-loop management system. Supported by an information-based traceability platform, this approach ensured strict monitoring and real-time correction across every stage from recovery to distribution, thereby maintaining sterility and preventing cross-contamination at its source. In addition, standardized procedures and continuous staff training reinforced the reliability of execution. Repeated SOP-based training and assessments enhanced technical proficiency, quality awareness, and accountability, ensuring the accurate implementation of infection prevention measures and reducing human error-related infection risks. Consistent with the present findings, Zheng J et al.^18^ and Werner G et al.^19^ also reported that implementing quality control-oriented nursing management strategies in CSSD markedly improves instrument processing quality while reducing operational errors and adverse management events.

### Limitations:

A small number of samples is the limitation of the study. In view of this, more samples should be included in future studies to further validate the findings of this study.

## CONCLUSIONS

The application of WPQC management in CSSD nursing practice may significantly improve qualification rates across all stages of instrument disinfection and sterilization, reduce error rates and nosocomial infection incidence, shorten processing time, and enhance compliance with standardized nursing practices, thus worthy of clinical promotion and implementation.

### Authors’ Contributions:

**FW:** Performed the studies, data collection, drafted the manuscript, is responsible and accountable for the accuracy or integrity of the work.

**NZ:** Performed the statistical analysis, study design.

All authors have read and approved the final manuscript.
